# OA16.01. Patients, physicians, and CAM providers regard communication as central for integrating conventional and CAM therapies for chronic pain

**DOI:** 10.1186/1472-6882-12-S1-O62

**Published:** 2012-06-12

**Authors:** C Ritenbaugh, L Penney, L DeBar, D Welch, J Schneider, C Catlin, A Firemark, C Elder

**Affiliations:** 1 University of Arizona, Tucson, USA; 2 Kaiser Permanente Center for Health Research, Portland, USA

## Purpose

Interviews were conducted in a large managed care organization (MCO) to explore the integration of community CAM services (acupuncture and chiropractic) with conventional care for chronic pain patients.

## Methods

Primary care providers (PCPs) were recruited, stratified on their rates of patient referral to CAM therapies. Acupuncturists and chiropractors were recruited from among those who treated MCO patients, stratified on numbers of MCO patients seen. Patients were recruited from among 11,960 respondents to a questionnaire on use of acupuncture and chiropractic for chronic pain, stratified on patterns of acupuncture and chiropractic use.

Providers (CAM and conventional) participated in individual qualitative interviews. Patients participated in focus groups and individual interviews following similar structured guides. Audio from interviews and focus groups were transcribed, and coded. This poster addresses communication and interaction among PCPs, CAM providers, and patients.

## Results

Interviews with 48 individuals and 11 focus groups were conducted between August 2011 and February 2012. The 90 patients were 78% female, average age 67.7 years, 71% white. Providers included 6 chiropractors, 7 acupuncturists, 8 family medicine clinicians, and 15 internists.

Most PCPs expressed frustrations with existing chronic pain treatment and desired more accessible treatment options. However, unknowns surrounding CAM modalities, and little feedback from patients on their responses to CAM therapies, created caution in referring chronic pain patients for CAM treatment. Patients consistently wanted more communication between their PCPs and CAM providers. CAM providers wanted a higher degree of communication or interaction with PCPs, specifically to coordinate care. All those interviewed (PCPs, patients, and CAM providers) wanted more systematic information about the availability and quality of CAM services and practitioners.

## Conclusion

Many PCPs are interested in and willing to refer their patients to CAM providers. However, PCPs, patients and CAM providers all identify the lack of communication and interaction as barriers to integrated patient care.

